# Prophylactic antenatal N-Acetyl Cysteine administration combined with postnatal administration can decrease mortality and injury markers associated with necrotizing enterocolitis in a rat model

**DOI:** 10.1371/journal.pone.0233612

**Published:** 2020-06-01

**Authors:** Osnat Zmora, Ola Gutzeit, Linoy Segal, Sari Boulos, Zvika Millo, Yuval Ginsberg, Nizar Khatib, Fadwa Dabbah-Assad, Ofer Fainaru, Zeev Weiner, Ron Beloosesky

**Affiliations:** 1 Department of Pediatric Surgery, Shamir Medical Center, Zerifin, Israel; 2 Department of Obstetrics and Gynecology, Rambam Medical Center, Haifa, Israel; Hopital Robert Debre, FRANCE

## Abstract

**Background:**

Necrotizing enterocolitis (NEC) is a devastating gastrointestinal disease of neonates, especially premature neonates. To date, there is no prophylactic treatment against NEC, except breast milk and slow increase in enteral feeding, and there is no antenatal prophylaxis.

**Aims:**

To assess possible protective effects of antenatal N-Acetyl Cysteine (NAC) against the intestinal pathophysiological changes associated with NEC in a rat model of NEC and against its associated mortality.

**Methods:**

Newborn Sprague-Dawley rats were divided into 5 groups: control (n = 33); NEC (n = 32)-subjected to hypoxia and formula feeding for 4 days to induce NEC; NEC-NAC (n = 34)-with induced NEC and concomitant postnatal NAC administration; NAC-NEC (n = 33)-born to dams treated with NAC for the last 3 days of pregnancy starting at gestational age of 18 days, and then subjected to induced NEC after birth; NAC-NEC-NAC (n = 36)—subjected to induced NEC with both prenatal and postnatal NAC treatment. At day of life 5, weight and survival of pups in the different groups were examined, and pups were euthanized. Ileal TNF-α, IL-6, IL-1β, IL-10, NFkB p65, iNOS and cleaved caspase 3 protein levels (western blot) and mRNA expression (RT-PCR) were compared between groups.

**Results:**

Pup mortality was significantly reduced in the NAC-NEC-NAC group compared to NEC (11% vs. 34%, P<0.05). Ileal protein levels and mRNA expression of all injury markers tested except IL-10 were significantly increased in NEC compared to control. These markers were significantly reduced in all NAC treatment groups (NEC-NAC, NAC-NEC, and NAC-NEC-NAC) compared to NEC. The most pronounced decrease was observed in the NAC-NEC NAC group.

**Conclusions:**

Antenatal NAC decreases injury markers and mortality associated with NEC in a rat model. Antenatal administration of NAC may present a novel approach for NEC prophylaxis in pregnancies with risk for preterm birth.

## Introduction

Necrotizing enterocolitis (NEC) is the leading gastrointestinal disease of the neonate affecting 3,000–5000 neonates in the US each year [1]. It affects mainly premature infants [2], with mortality rate as high as 30% [3,4]. The involved bowel wall demonstrates inflammatory infiltration with bowel necrosis [5,6], increased intestinal cells apoptosis [7–10], altered levels of cytokines [6,7,11], and Increased oxidative stress [8,12,13]. Various biochemical markers have been reported to be involved in NEC in human and in animal studies, including TNF-α and IL-6 [6,14,15], IL1- β [16], IL-10 and NFkB [14,15], iNOS [7,8,17]) and caspase 3 [9]. Prevention measures are scarce [18]. Gentle enteral feeding with preferably breast milk is the only widely used prevention [19,20]. Although clinical data support the addition of probiotics for prevention [21,22], this method has not gained popular use due to fears of increase in sepsis incidents [23], and currently, there is only a conditional recommendation to provide specific strains in order to reduce NEC rate [24].So far, there are no published studies investigating prophylactic treatment to mothers at risk for preterm labor for the prevention of NEC. Currently, there are few indications for prophylactic antenatal treatment to mothers at risk for preterm labor, mostly maternal steroids for the prevention of neonatal respiratory distress syndrome [25] and Magnesium Sulphate for the prevention of neonatal brain injury[26].

N-Acetyl Cysteine (NAC) is a known anti-oxidant and anti-inflammatory agent. It is widely used for paracetamol intoxication, and is considered safe for use during pregnancy (class B) [27].A human study demonstrated rapid transfer of NAC from the mother to the fetus through the placenta with umbilical cord concentrations frequently exceeding maternal concentrations [28]. NAC has been demonstrated to attenuate fetal and neonatal inflammation and oxidative stress in different models of maternal inflammation both in animals [19, 24] and in humans [24]. There are two animal studies in the literature [29,30] describing successful offspring NAC administration for the treatment of NEC.

In the current study we used an established rat model of NEC [7,31] to study antenatal NAC prophylaxis for the prevention of NEC, and in combination with postnatal NAC treatment.

## Materials and methods

### Study groups

**Fig 1:**
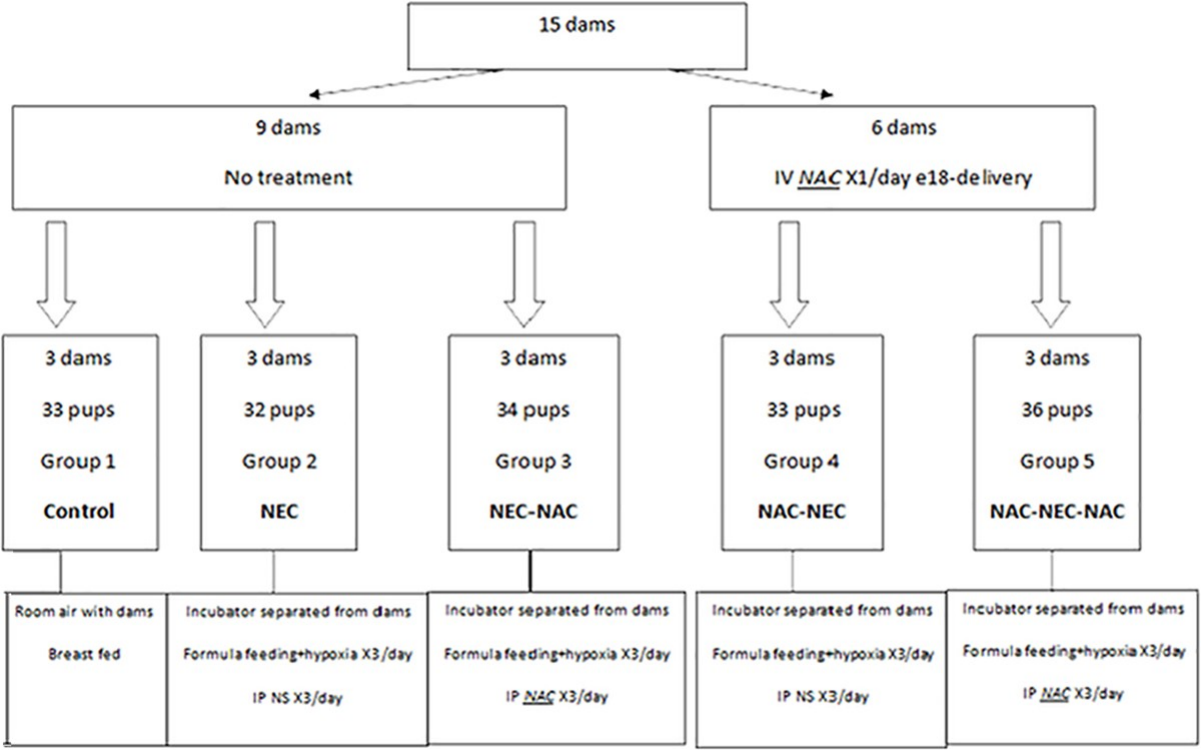
Flowchart of the different study groups. NAC- N-Acetyl Cysteine; NEC- necrotizing enterocolitis; NS-Normal. Saline; IP- Intra-Peritoneal.

Pregnant Sprague-Dawley rats were obtained from ENVIGO RMS (Israel) at gestational day 11 and were allowed to acclimate for 7 days prior to beginning of the experiments. Animals were maintained in temperature (25°C) and light controlled facilities with access to food and water ad libitum throughout the study. Fig 1 presents the allocation of the study groups. The first group of pregnant rats (9 dams) received no treatment and delivered spontaneously. After birth, the pups were divided into 3 groups:

#### Group 1- control

33 pups were left with their mothers (3 dams) and were breastfed.

#### Group 2 –NEC

32 pups from 3 other dams were transferred to an incubator (Ohio Medical Products, Madison, Wis). We used an established protocol of NEC induction [7,31] in which pups were fed thrice daily by gavage of 0.2 mL clean formula consisting of 15 g of Similac 60/40 (Abbott Nutritional, Columbus, OH) in 75 mL of Esbilac canine milk replacement (PetAg Inc., Hampshire, IL). The pups also received three times (10 minutes each) daily hypoxia exposure with 5% O_2_ and 95% N_2_ and intraperitoneal (IP) injections of saline following each NEC treatment.

#### Group 3- NEC-NAC

34 pups from the remaining three dams received the same conditions as pups in group 2, with NAC treatment (N-Acetyl-Cysteine, SIGMA, reconstituted in water, 300mg/kg IP) 3 times daily instead of saline, following each NEC treatment.

To explore the possible protective effect of NAC during pregnancy, a second group of 6 pregnant rats received IV injections of NAC (300mg/kg) once daily for the last three days of pregnancy (from gestational day 18 until gestational day 20, as delivery occurred at gestational day 21). After birth, the pups were randomly divided into groups 4 and 5 as follows:

#### Group 4- NAC-NEC

33 pups were separated from their dams and received the same conditions as group 2 (NEC) above.

#### Group 5: NAC-NEC-NAC

36 pups were separated from their dams and received the same conditions as group 3 above (NEC-NAC).

Pups in all groups were euthanized on the fifth day of life.

### Newborn weight, survival, and sample collection

All newborns were weighed after birth and on the fifth day of life before sacrifice. Mortality of pups by the fifth day was compared among the different treatment groups. On the fifth day of life pups were anesthetized with isoflurane and were decapitated. The distal 3 cm of terminal ileum were harvested and then immediately frozen in liquid nitrogen for further processing and analysis. Processing and analysis were performed on the mixture of cellular and intracellular components of the terminal ileum. Ileum protein levels were determined by Western blot, and RNA was extracted for RT-PCR analysis. Markers tested included some of the markers previously reported in the literature as involved in the inflammatory, oxidative stress and apoptotic injury pathways in NEC in both human and animals: TNF-α and IL-6 [6,14,15], IL1- β [16], IL-10 and NFkB [14,15], iNOS [7,8,17], and caspase 3 [9].

### Western blot analysis

Preparations of cell Lysates and Western blot cells or tissues were lysed in RIPA buffer (phosphate-buffered saline containing 1% Nonidet P-40, 0.1% sodium dodecyl sulfate [SDS], 1mM Na3VO1, 4 mM phenylmethylsulfonyl fluoride and 0.05% [w/v] aprotinin). Insoluble proteins were discarded by high-speed centrifugation at 4°C. Small volume of lysate was taken to perform protein estimation assay. Protein estimation was performed using absorbance at 280 nm in triplicate by Nanodrop. Determination of the protein concentration samples was also compared to standards (BSA), ensuring that the standard is diluted into the same buffer as the samples.

50 μg of each sample were loaded in each well of a 12% SDS-PAGE acrylamide gel.

After transferring and blocking to nitrocellulose membranes the blots were incubated over night with the primary antibodies against the target protein at 4°C. The antibodies were diluted in blocking buffer according to the manufactures recommended ratio.

Following the incubation with the primary antibodies and rinsing the blots, the blots were incubated for 1h at room temperature with HRP-conjugated secondary antibody (according to the manufactures recommended ratio-usually 1:10000).

Antibodies recognizing the nuclear factor kappa-light-chain-enhancer of activated B cells (NFkB) p65, active subunit (Merck, Millipore, Massachusetts), Inducible nitric oxide synthase (iNOS), Tumor necrosis factor-α (TNF-α), interleukin (IL)-6, IL-1β, IL-10 (Novus Biologicals, Centennial, CO) and the cleaved caspase 3 (Cleaved Caspase-3 (Asp175) (Novus Biologicals, Centennial, CO) were used in combination with a goat anti- rabbit and donkey anti- mouse horseradish peroxidase-conjugated secondary antibody (Jackson Immunoresearch Laboratories, West Grove, PA). We used actin as our housekeeping protein. Caspase-3 antibody detects endogenous levels of full length caspase-3 (35 kDa) and the large fragment of caspase-3 resulting from cleavage (17 kDa). Therefore, 2 bands of cleaved caspase 3 were detected.

### Densitometric analysis

In order to compare target protein expression levels between the different samples on the same blot or across blots we normalized the bands by loading actin as our housekeeping protein. We used a densitometer to scan and to measure the signal generated on a Western blot. Imaging software was used to compare the signal generated by the bands detected on the Western blot. The quantification reflects the relative amounts as a ratio of each protein band relative to the lanes of the control-the actin.

Blots were stripped and reprobed using Thermo Scientific Restore PLUS Western Blot Stripping Buffer (Catalog number: 46430)

### Gene expression analysis

#### RNA purification

RNA was extracted from fetal ileum by combining TRI reagent (Sigma-Aldrich, St. Louis, MO) and PureLink^TM^ kit (Thermo Fisher Scientific, Waltham, MA). TRI reagent aqueous upper phase was loaded on PureLink^TM^ Columns and purified.

#### Real Time PCR

cDNA was synthesized using the High Capacity cDNA Reverse Transcription kit (Thermo Fisher Scientific, Waltham, MA). Real-time PCR was performed using Sybr Green FastMix, ROX (Quanta Bioscience, Gaithersburg, MD) and CORBETT (ilex, Medical ltd). The thermal cycling program was as follows: hold on 95°C for 3 min followed by 40 cycles of: 10 s at 95°C and 60 s at 60°C. The relative expression of,TNF-α, iNOS, IL1- β, IL-10, NFkB, IL-6 and caspase 3 was normalized to β-actin, and calculated using the ΔΔCt method.

### Statistical analysis

Number of pups (median and 25%-75% range) and weight of pups (mean ± SD) were compared between groups 1-2-3 (born to dams with no prenatal treatment) and groups 4–5 (born to dams which received antenatal NAC). Terminal ileum caspase 3, NFkB p65, iNOS, TNFα, IL-6, L-1β, and IL-10 protein and MRNA expression levels were compared between pups from the different groups (N = 5 ileum samples from each group). All results were expressed as means ± SD using one-way analysis of variance followed by post hoc tests for pairwise comparisons (Holm-Sidak method). Differences were considered to be significant at *P<* 0.05. Sigma Stat software version 4.0 was used to perform statistical analysis. All methods were performed in accordance with the relevant guidelines and regulations.

### Ethical approval

This study was carried out in strict accordance with the recommendations in the guide for care and use of laboratory animals of the Israeli national institute of health. The protocols and procedures were approved by the Institutional Animal Care Committee at the Rappaport Research and Education Institute (Protocol number: IL001-01-2016). Guidelines for the care and use of the animals approved by the local institution were followed. All efforts were made to minimize animal suffering. The study was supervised by a veterinarian on a daily basis. The pain/ suffering of the rats were categorized as low. Humane endpoints were: weight loss more than 20%, sepsis, severe bloody diarrhea, and no food intake. The rats were monitored daily for these humane endpoint

## Results

### Number and weight of newborn pups

There were no differences in the litter size or newborn pup weight on day 1 between groups 1-2-3 versus the groups 4–5. On day of life 5, the average weight of the control pups was significantly higher compared to all other groups. The average weight of pups from group 5 (NAC-NEC-NAC) was significantly higher than in groups 2 (NEC) and 3 (NEC-NAC), and similar to group 4 (NAC-NEC) (Table 1).

**Table 1:** Clinical variables of study groups.

Variable Group	Control	NEC	NEC-NAC	NAC-NEC	NAC-NEC-NAC
**Litter size**^**a**^	12.5 (10–14)			11 (10–13)	
**Weight day 1**	6.5±0.6 gr			6.7±0.5 gr	
**Weight day 5**	10+0.9 gr*^	6+0.6 gr	6+0.5 gr	6.2+0.8 gr	6.57+0.7 gr*
**Mortality**	0% (0/33) *^	34% (11/32)	26% (9/34)	24% (8/33)	11% (4/36)*

^**a**^Litter size is expressed as median and interquartile range (25^th^-75^th^).

*Significant difference from NEC group, *P*<0.05.

^Significant difference from all other groups.

### Mortality of pups

NEC was associated with pup mortality of 34% by the fifth day of life (11/32), while NAC treatment to both dams and pups (NAC-NEC-NAC) significantly reduced pup mortality to 11% (4/36) (*P*<0.05). Mortality rates of pups in when NAC was only administered to dams (NAC-NEC) (24%, 8/33) or only to offspring (NEC-NAC) (26%, 9/34) were not significant different from NEC group. There were no mortalities in the control group (Table 1).

### Ileal protein levels

**Fig 2:**
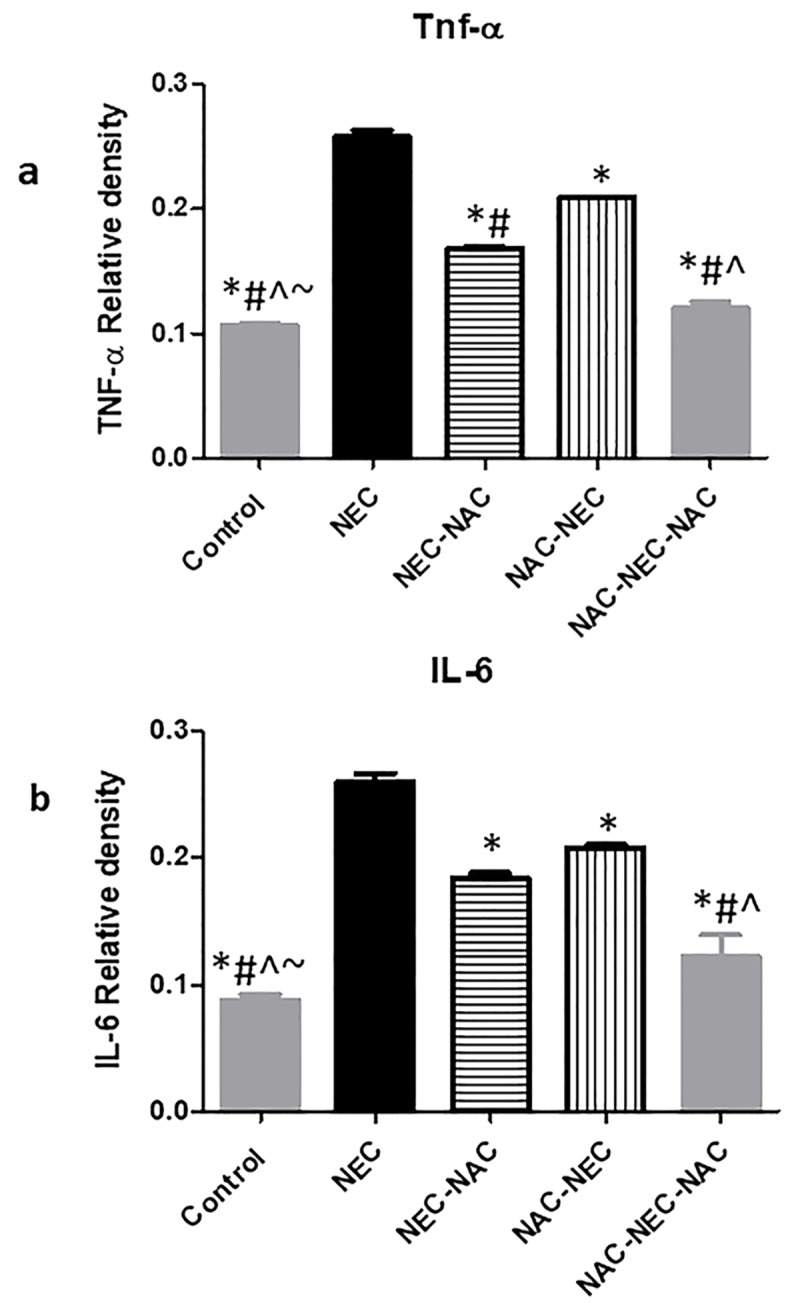
Ileal TNFα and IL-6 protein levels.

**Fig 3:**
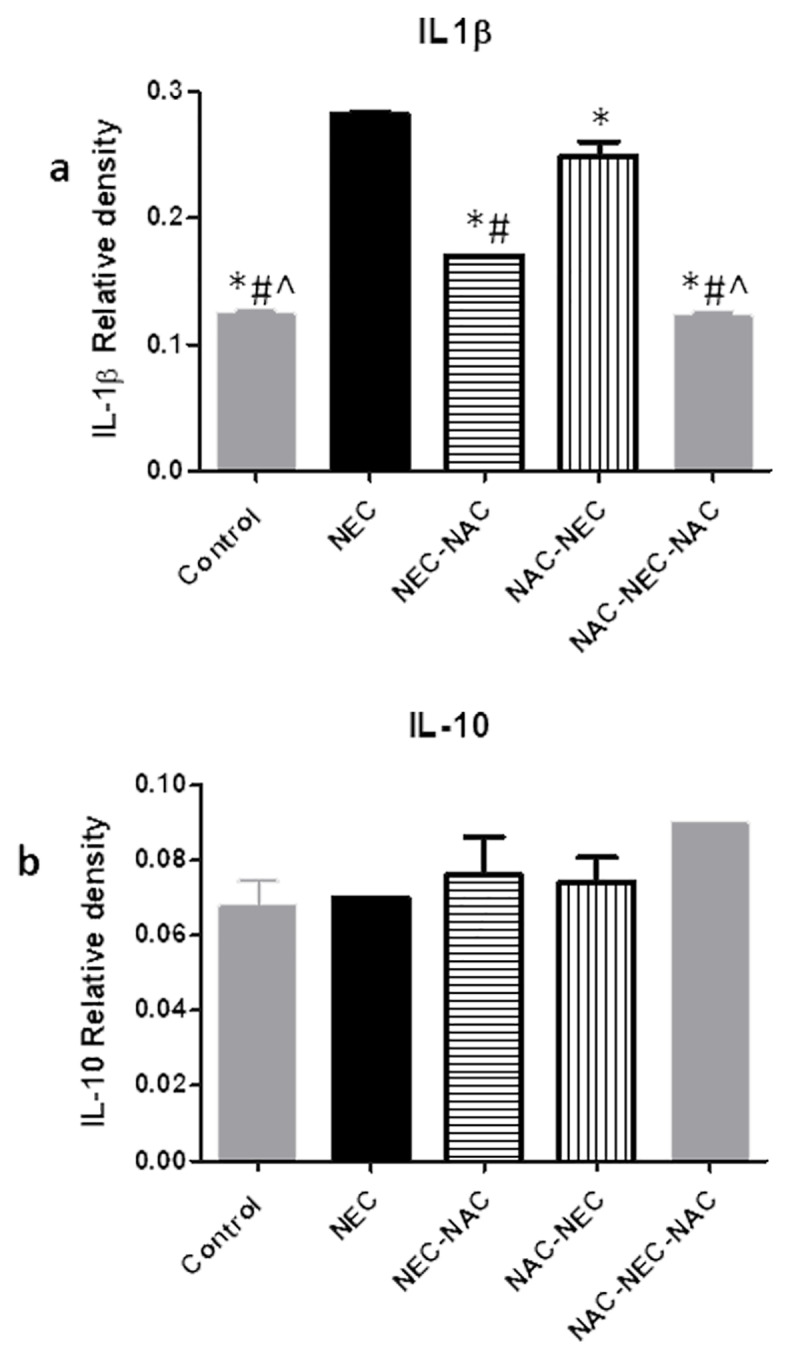
Ileal IL-1β and IL-10 protein levels.

**Fig 4:**
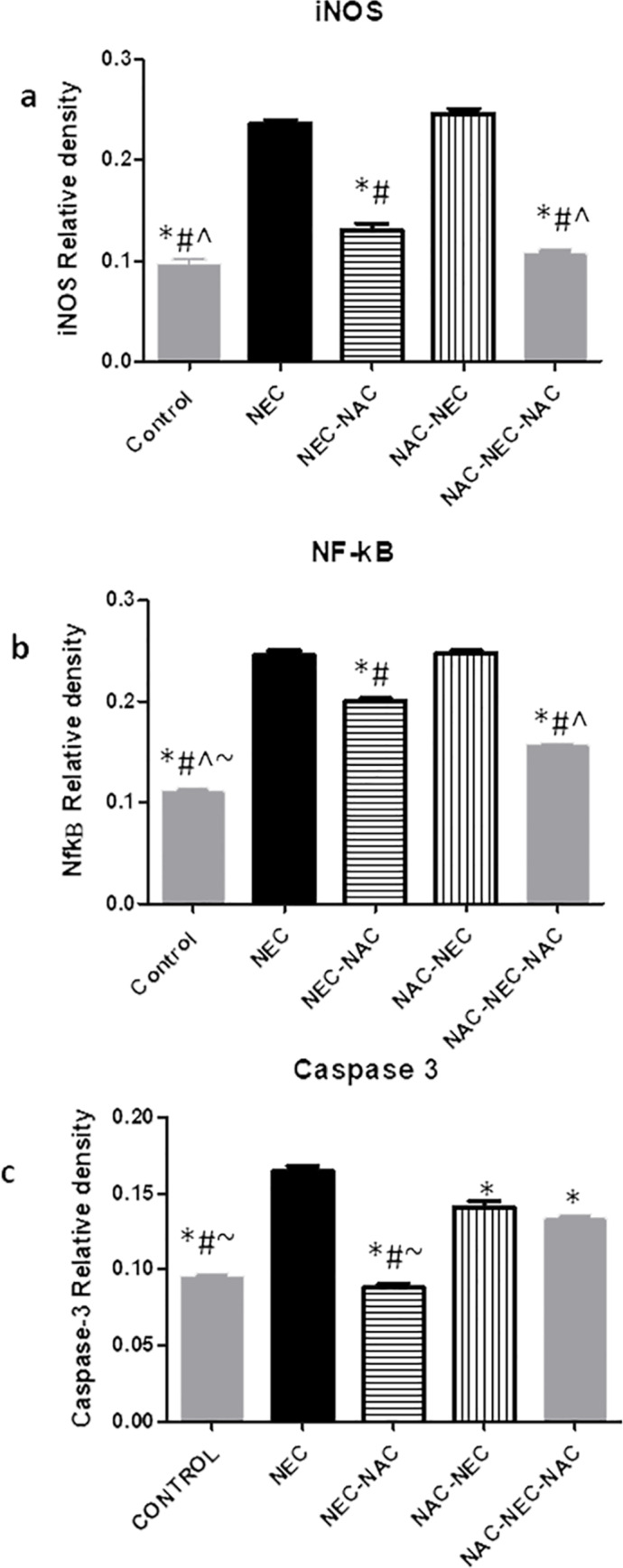
Ileal p65 (NF-κB), iNOS and caspase 3 protein levels*. Significant difference (p<0.05) from NEC. # Significant difference (p<0.05) from NAC-NEC. ^ Significant difference (p<0.05) from NEC-NAC. ~ Significant difference (p<0.05) from NAC-NEC-NAC.

NEC pups had significantly increased ileal inflammatory protein levels of pro inflammatory cytokines TNFα, IL-6, IL-1β and increased protein levels of NFkB active unit, iNOS (pathway activation) and cleaved caspase 3 compared to controls. No change was demonstrated in IL-10 protein levels among the different groups (Figs 2–4, Table 2).

**Table 2:** Marker protein and mRNA levels in the different groups.

	Control	NEC	NEC-NAC	NAC-NEC	NAC-NEC-NAC
**TNFα**					
Protein	0.1±0.005*	0.25±0.01	0.17±0.004*	0.21±0.001*	0.12±0.012*
mRNA	1.08±0.02*	2.05±0.06	1.24±0.06*	1.84±0.08*	1.23±0.07*
**IL-6**					
Protein	0.09±0.01*	0.26±0.01	0.18±0.009*	0.21±0.005*	0.12±0.035*
mRNA	1.09±0.02*	2.01±0.08	1.94±0.06*	1.8±0.1*	1.19±0.07*
**IL-1β**					
Protein	0.12±0.005*	0.28±0.004	0.17±0.001*	0.25±0.027*	0.12±0.008*
mRNA	1.12±0.07*	1.97±0.09	1.24±0.05*	1.77±0.05*	1.23±0.04*
**IL-10**					
protein	0.07±0.014	0.07±0.001	0.08±0.023	0.07±0.015	0.09±0.001
mRNA	1.05±0.032	1.05±0.029	1.07±0.034	1.07±0.031	1.05±0.024
**NFkB**					
protein	0.11±0.007*	0.25±0.008	0.2±0.007*	0.25±0.004	0.16±0.005*
mRNA	1.11±0.05*	1.6±0.15	1.37±0.05*	1.31±0.08*	1.25±0.04*
**iNOS**					
protein	0.1±0.011*	0.24±0.008	0.13±0.015*	0.25±0.011	0.1±0.011*
mRNA	1.13±0.04*	1.76±0.12	1.4±0.08*	1.44±0.08*	1.15±0.04*
**Caspase 3**					
protein	0.1±0.005*	0.17±0.007	0.09±0.005*	0.14±0.008*	0.14±0.006*
mRNA	1.18±0.05*	1.77±0.15	1.28±0.04*	0.16±0.046	1.24±0.06*

*P<0.001

NAC administration to dams only during pregnancy (NAC-NEC) was associated with a significant decrease in ileal proinflammatory cytokines TNFα, IL-6, IL-1β and in protein levels of cleaved caspase 3 compared to NEC. There was no change in protein levels of NFkB active unit, iNOS and IL-10. The same pattern was demonstrated when NAC was administered only to the offsprings (NEC-NAC), except the reduction that was demonstrated in this group in NFkB and in iNOS protein levels. The most pronounced decrease in all protein levels except caspase 3 was demonstrated within the NAC-NEC-NAC group where NAC was administered both to dams and offsprings (Figs 2–4) (Table 2).

### mRNA expression

**Fig 5:**
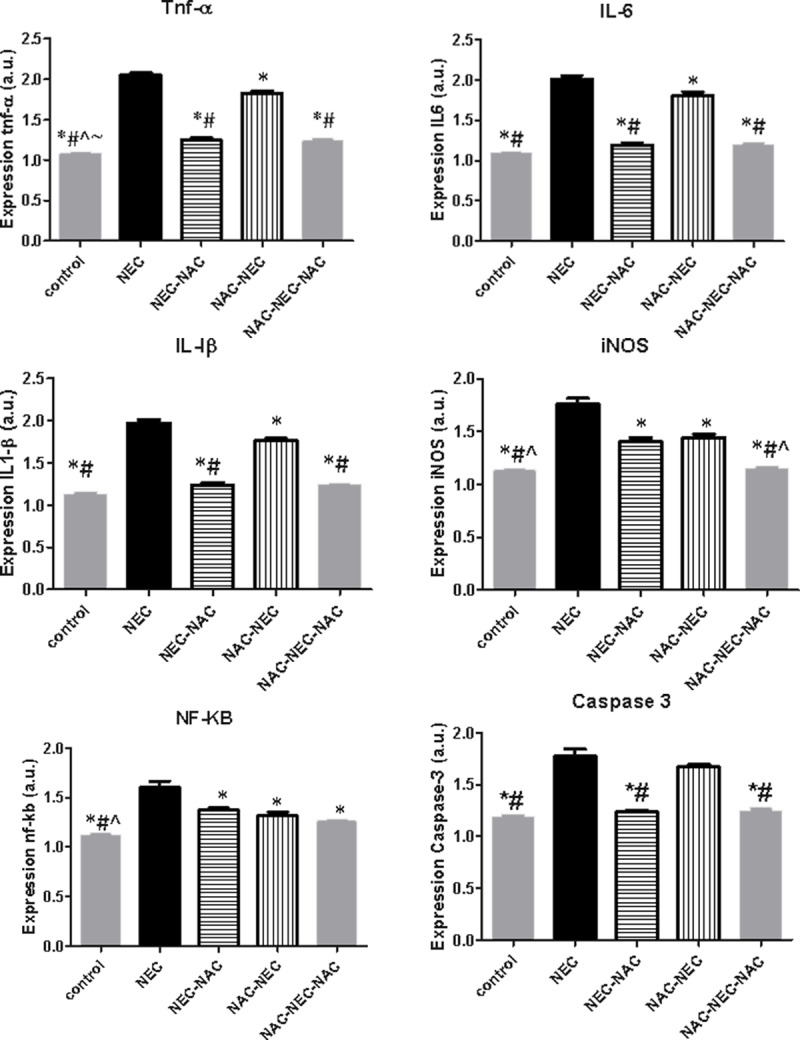
Ileal inflammatory mediators mRNA expression. * Significant difference (p<0.05) from NEC. # Significant difference (p<0.05) from NAC-NEC. ^ Significant difference (p<0.05) from NEC-NAC. ~ Significant difference (p<0.05) from NAC-NEC-NAC.

NEC pups had significantly increased mRNA expression of ileal pro-inflammatory mediators TNFα, IL-6, IL-1β and increased expression of NFkB active unit, iNOS (pathway activation) and cleaved caspase 3 compared to controls (Fig 5). No change was demonstrated in IL-10 mRNA expression among the different groups (Table 2).

In all NAC treatment groups (NAC-NEC, NEC-NAC, NAC-NEC-NAC) the expression was decreased compared to NEC pups) Fig 5).

## Discussion

Prenatal antepartum administration of NAC to dams and/or postnatal administration to pups exposed to NEC conditions significantly decreased ileal TNFα, IL-6, IL-1β, NFkB active unit, iNOS, and cleaved caspase 3 protein levels and mRNA expression. Furthermore, NAC administration to both dams and offsprings decreased mortality and increased the offspring weight compared to NEC pups.

Bowel injury in NEC is associated with both increased gut oxidative stress and excessive activation of the innate immune response through TLR-4 and NFkB pathways in response to LPS. These changes can lead to injury and loss of enterocytes through apoptosis [4,6,10,17]. In our experiments, we have demonstrated that specific biochemical markers which have been previously reported to be involved in NEC can be decreased by NAC.

NAC is a known anti-oxidant and anti-inflammatory agent. Its anti-inflammatory activity is mediated through inhibition of NF-kB and attenuation of pro-inflammatory cytokine synthesis [32,33]. It has been previously described in two animal studies [29,30] as a successful treatment for NEC when administered to offsprings. NAC has also been widely studied as treatment against maternal and resultant fetal and newborn inflammation in various animal models [34,38]. Previous studies have demonstrated that when NAC alone is given to pregnant rats, maternal as well as fetal plasma levels of IL-6, IL-1β, and IL-10 are unchanged compared to control groups in which only saline was injected [34]. However, in a rat model of maternal inflammation, maternal NAC administration decreased both maternal and fetal serum pro inflammatory protein levels [34]. In humans, the administration of NAC decreased NF-κB activation in mononuclear leukocytes, which was associated with a decrease in IL-8 in patients with sepsis [35]. In a randomized controlled double-blind study in humans, 22 pregnant patients diagnosed with chorioamnionitis were randomized with their infants to NAC or saline treatment. Infants treated with NAC had higher serum anti-inflammatory interleukin-1 receptor antagonist level versus controls [36].

The anti-oxidant activity of NAC is inflicted by its conversion into metabolites that are capable of stimulating glutathione synthesis, promoting detoxification, and by its own function as an excellent scavenger of free radicals [37,38]. The anti-oxidative properties of NAC with induction of this protection from the mother to the fetus were demonstrated by Buhimschi et al. In a maternal inflammation model in mice, maternal inflammation resulted in oxidative stress associated with maternal and fetal liver glutathione (GSH) precursor depletion, while maternal NAC restored both maternal and fetal oxidative balance and increased liver GSH levels in both dams and fetuses [38].

NAC is also widely used for paracetamol intoxication, and is considered safe for use during pregnancy (class B) [27]. These were additional reasons for choosing NAC as a candidate for prophylactic administration during pregnancy. In general, there are few indications for prophylactic antenatal treatment to mothers at risk for preterm labor, mostly maternal steroids for the prevention of neonatal respiratory distress syndrome [25] and Magnesium Sulphate for the prevention of neonatal brain injury [26].

In the current study we demonstrated that when NAC was administered to dams during pregnancy, its effects were preserved in the neonatal period. When the offsprings were exposed to NEC conditions, they were resilient to the bowel injury observed in offsprings which were exposed to the same conditions but without prior NAC prophylaxis given to dams. Interestingly, we demonstrated that prophylactic treatment administered to dams may be as effective as treatment given to the offsprings. The most pronounced decrease in intestinal injury was demonstrated within the NAC-NEC-NAC group. Maternal NAC followed by NAC to the offsprings had a cumulative effect on the bowel. Our novel finding that maternal NAC may protect the offsprings from future NEC is supported by a human study that demonstrated rapid transfer of NAC from the mother to the fetus through the placenta with umbilical cord concentrations frequently exceeding maternal concentrations [28]. This may prepare the newborn to the possible NEC insults and attenuate the inflammatory and oxidative responses associated with NEC.

In humans, NEC is associated with a mortality rate of 30% [3,4]. In our animal studies the mortality rate was 34%. Maternal NAC followed by NAC to offsprings decreased mortality dramatically to 11%. A previous study using a mice inflammation model demonstrated similar results. When NAC was administered to dams in cases of systemic inflammation in pregnancy, it decreased fetal mortality rate compared to fetuses exposed to maternal inflammation without NAC [38].

Our study is limited as it is an animal study with little clinically correlated data except for pup weight and mortality rate at the conclusion of the experiments. It should be remembered that the injury markers tested are not routinely monitored in clinical settings to diagnose and follow up on NEC presentation and evolution in human premature babies. Therefore, careful clinical and human studies are needed before drawing any clinically relevant conclusions.

## Conclusion

We have demonstrated in a rat NEC model that antenatal maternal NAC administration, especially when combined with postnatal administration, can decrease levels of biochemical markers associated with inflammatory changes, oxidative stress and apoptosis in the bowel of neonates exposed to NEC conditions, with an associated reduction in mortality.

Further studies are needed to evaluate the role of NAC in humans when there is an increased risk for the development of NEC, such as in anticipated preterm births <1500 grams.

## Supporting information

S1 Fig Uncropped and unadjusted images underlying all blot or gel results. (PDF)

S1 Table Reference primers. (DOCX)

S1 Data (PDF)
