# Supplementary material for: Prophylactic antenatal N-Acetyl Cysteine administration combined with postnatal administration can decrease mortality and injury markers associated with necrotizing enterocolitis in a rat model
Source: PLoS One. 2020 Jun 1;15(6):e0233612. doi: 10.1371/journal.pone.0233612 (PMC7263616; doi:10.1371/journal.pone.0233612)
Supplement: S1 Fig — (PDF) [file pone.0233612.s001.pdf]

Actin
FIG 2-4

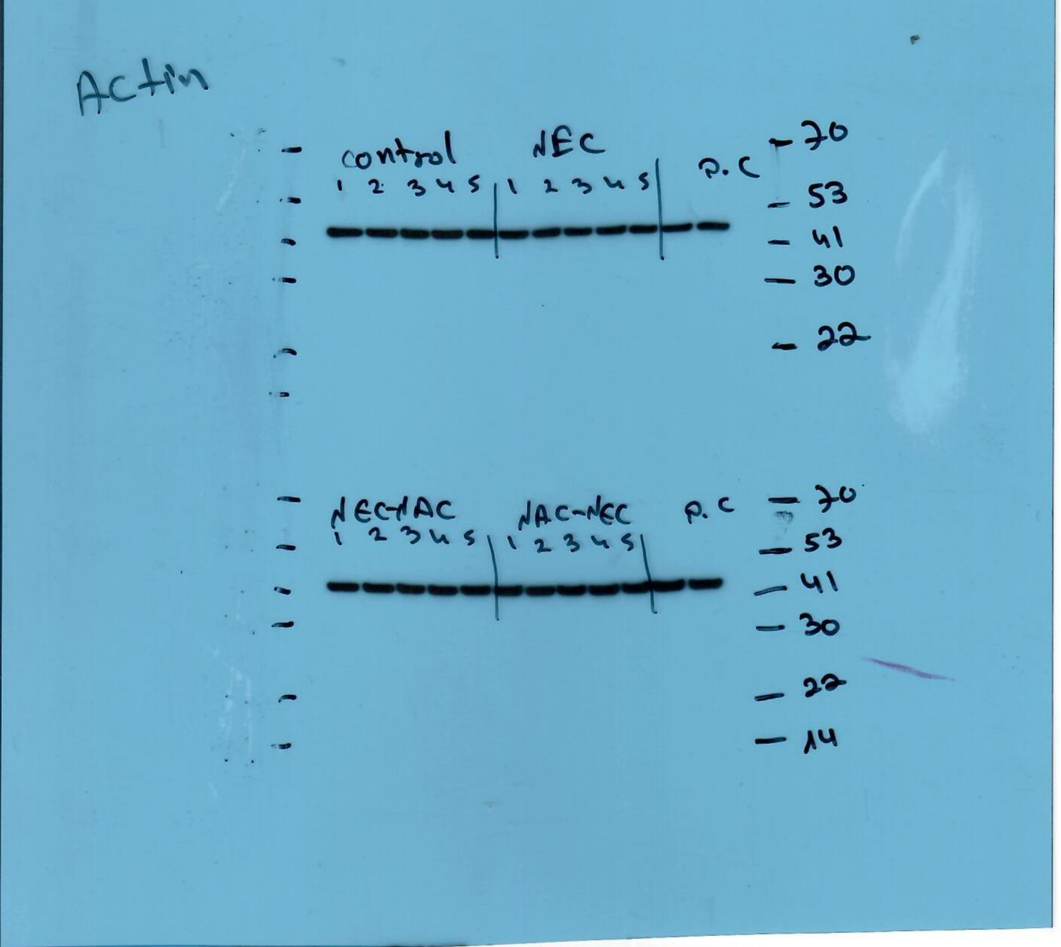
Actin
control NEC
1 2 3 4 5 1 2 3 4 5
P.C
70
53
41
30
22
NEC-NAC NAC-NEC
1 2 3 4 5 1 2 3 4 5
P.C
70
53
41
30
22
14

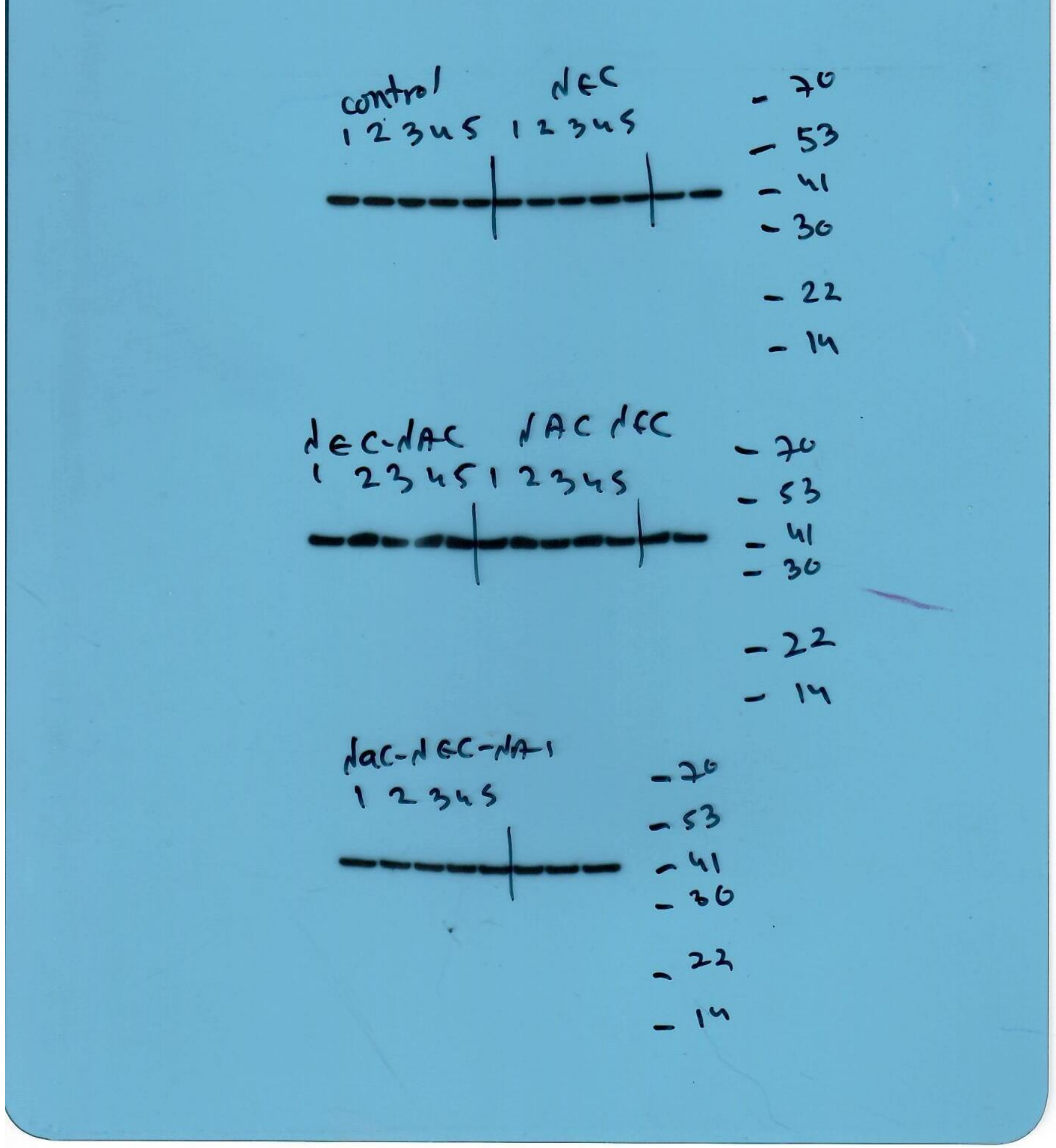
control NEC
1 2 3 4 5 1 2 3 4 5
70
53
41
30
22
14
NEC-NAC NAC NEC
1 2 3 4 5 1 2 3 4 5
70
53
41
30
22
14
1 2 3 4 5
70
53
41
30
22
14

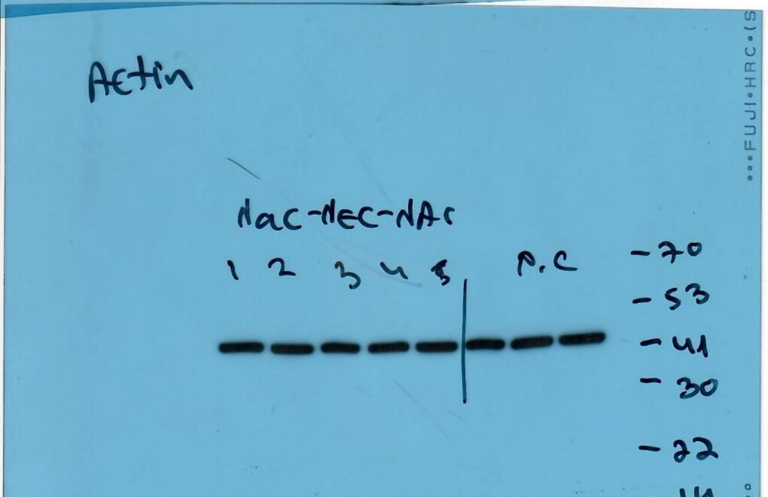
Actin
NAC-NEC-NAC
1 2 3 4 5
P.C
70
53
41
30
22

Actin
FIG 2-4

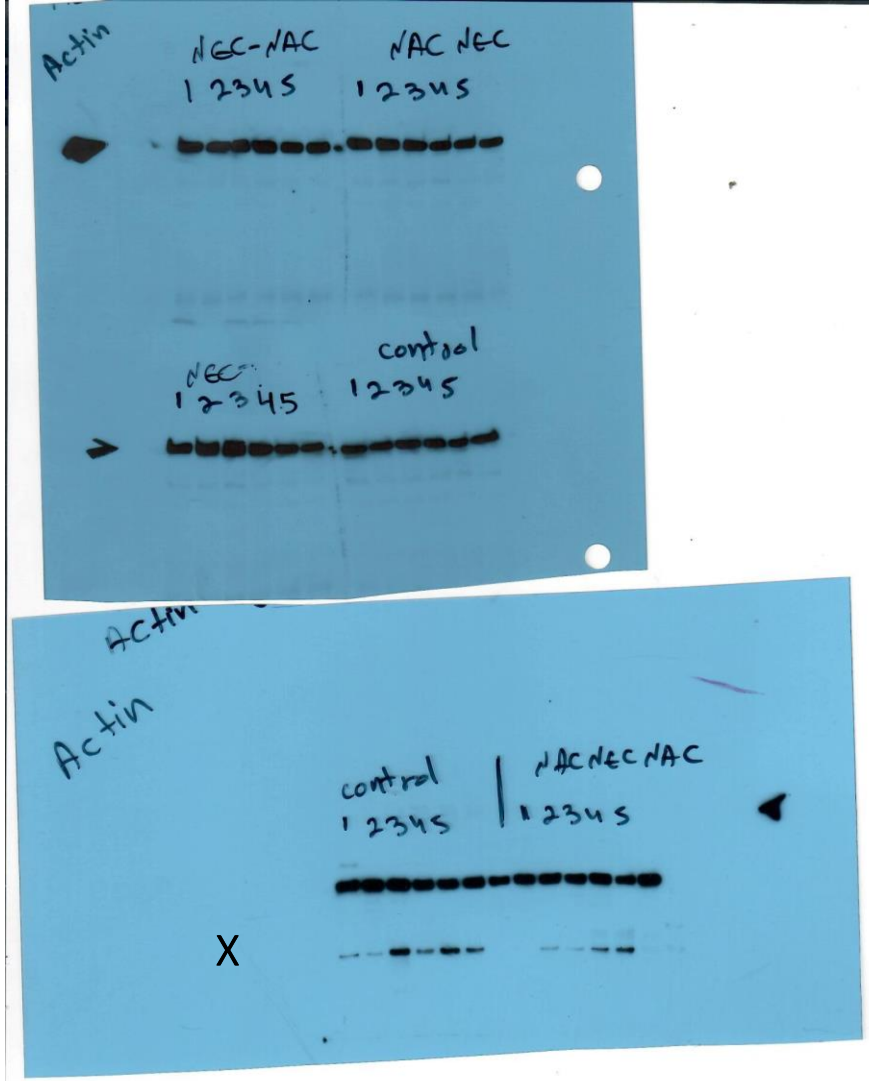

Actin
NEC-NAC
NAC NEC
1 2 3 4 5
1 2 3 4 5
NEC
1 2 3 4 5
control
1 2 3 4 5
Actin
control
1 2 3 4 5
NAC NEC NAC
1 2 3 4 5
X

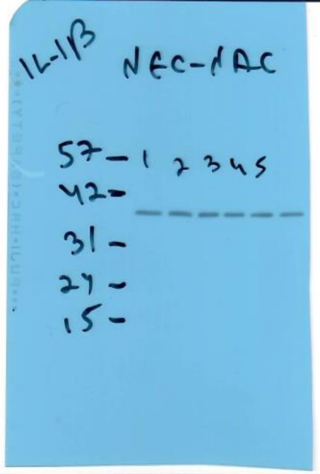
IL-1B
NEC-NAC
57 42 31 27 15
1 2 3 4 5

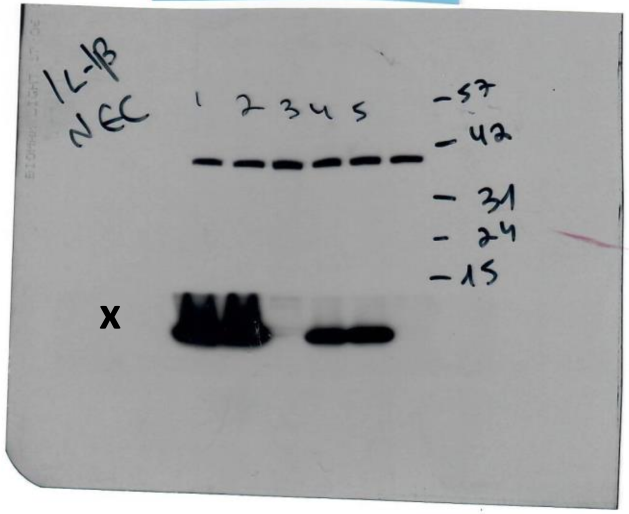
IL-1B
NEC
1 2 3 4 5
-57 -42 -31 -24 -15
x

IL-1ß

Fig 3 A

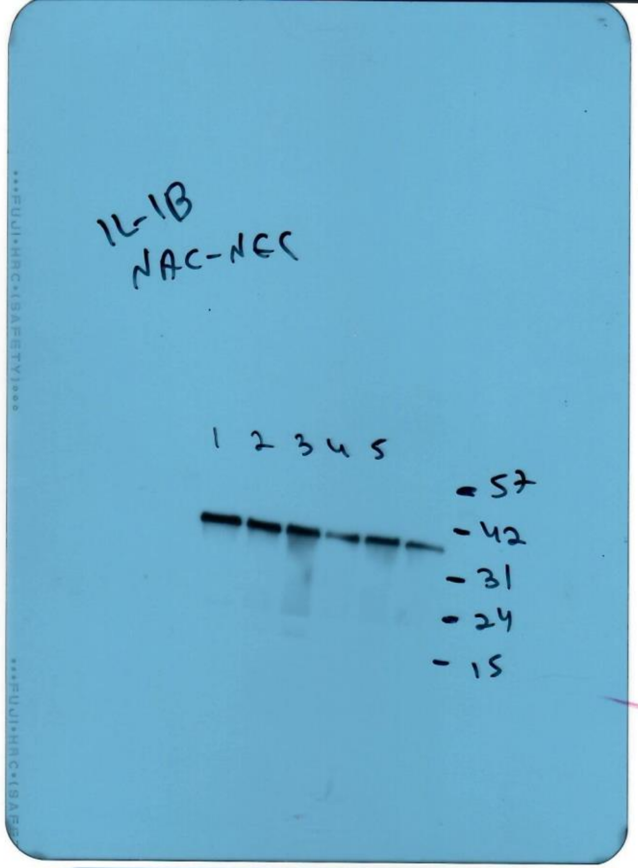
IL-1B
NAC-NEC
1 2 3 4 5
57 42 31 24 15

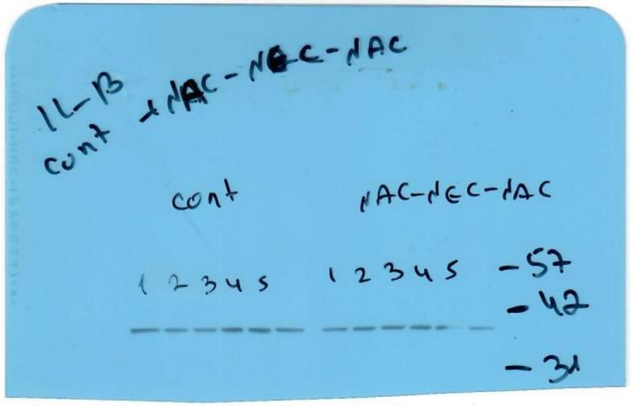
IL-1B
cont
cont
NAC-NEC-NAC
1 2 3 4 5
1 2 3 4 5
-57 -42 -31

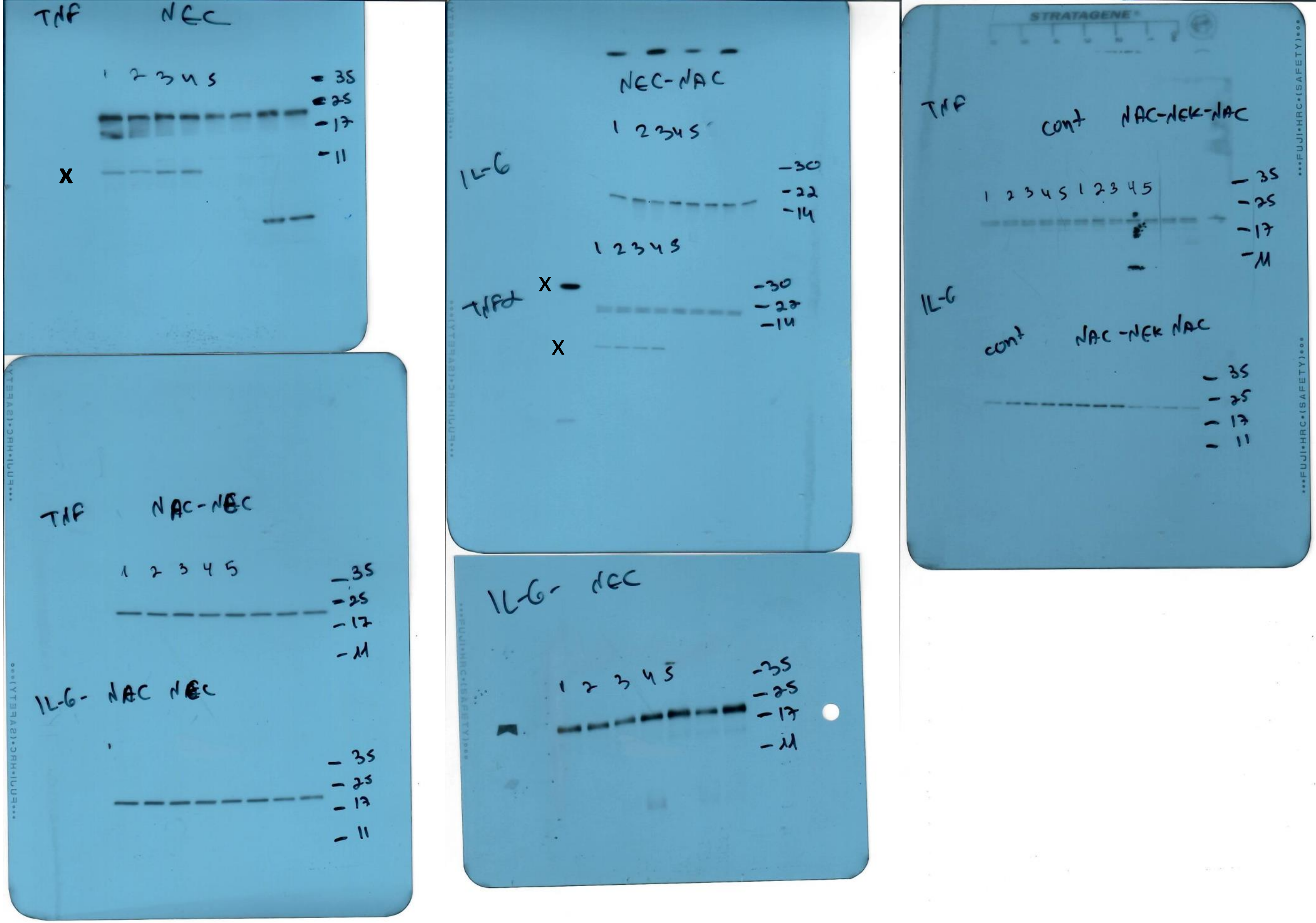

TNF+IL-6
FIG 2 A B

CASPASE 3

FIG 4 C

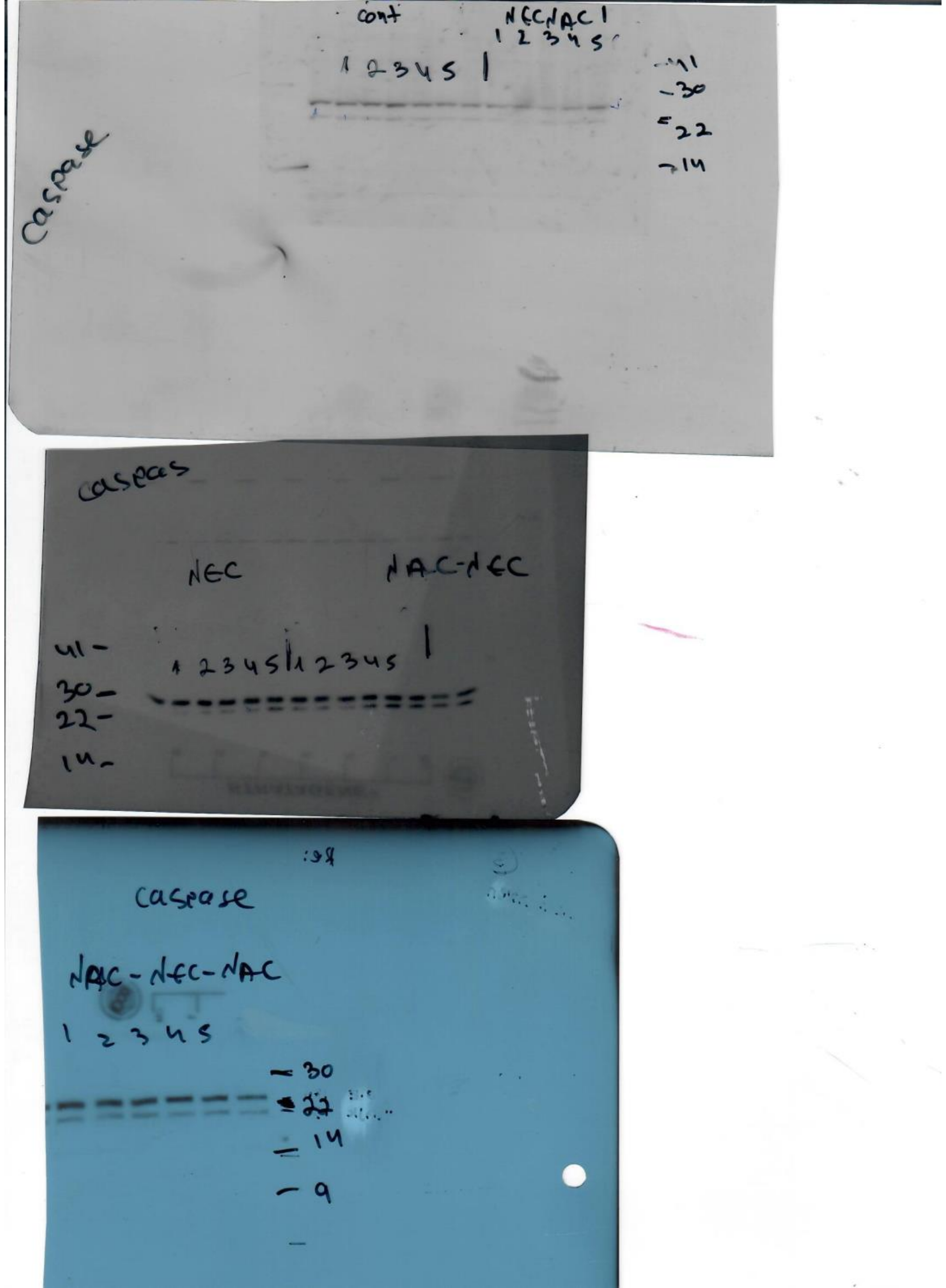

IL-10
FIG 3 B

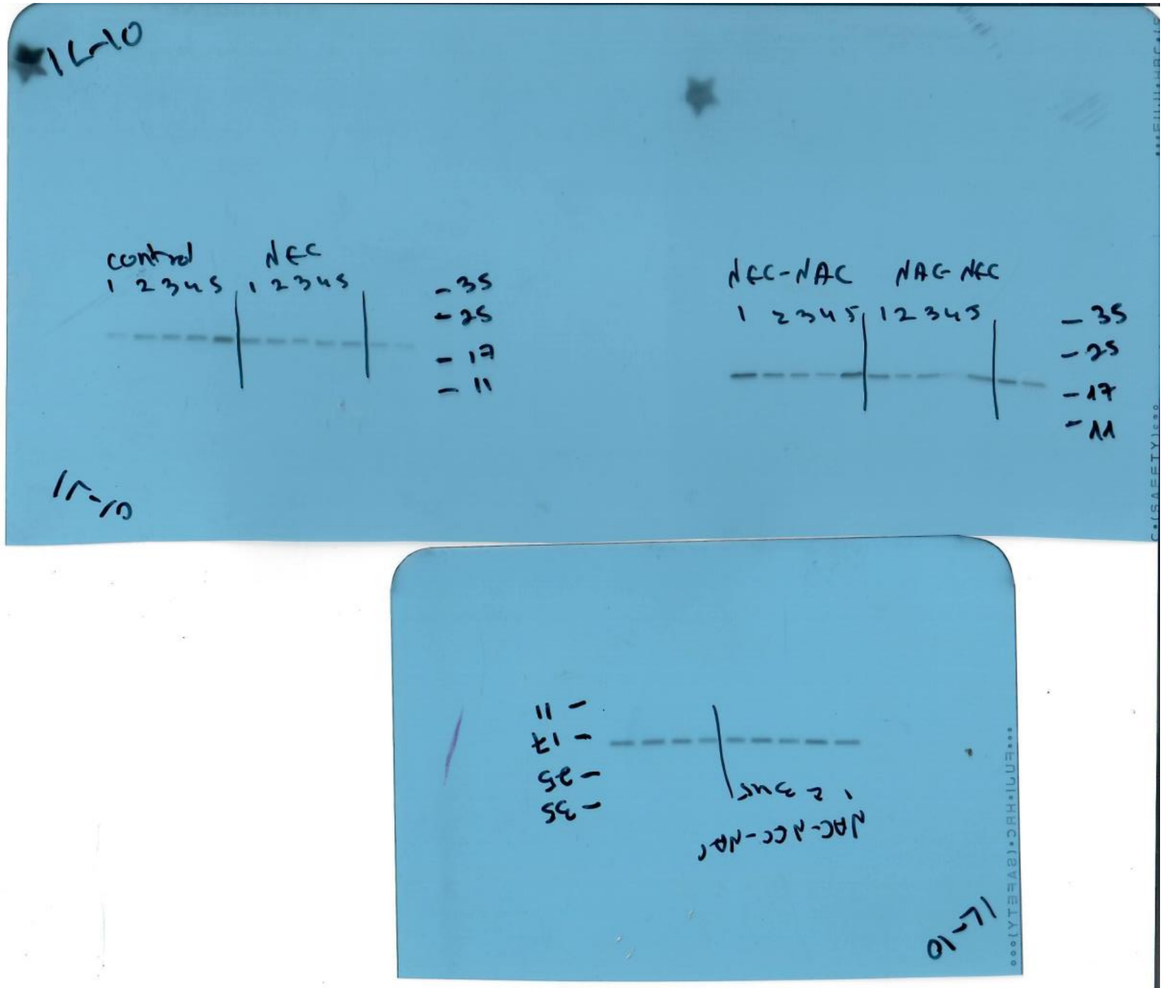

IL-10
control
NEC
1 2 3 4 5
1 2 3 4 5
35
25
17
11
NEC-NAC
NAC NEC
1 2 3 4 5
1 2 3 4 5
35
25
17
11
IL-10

Inos
Fig 4 A

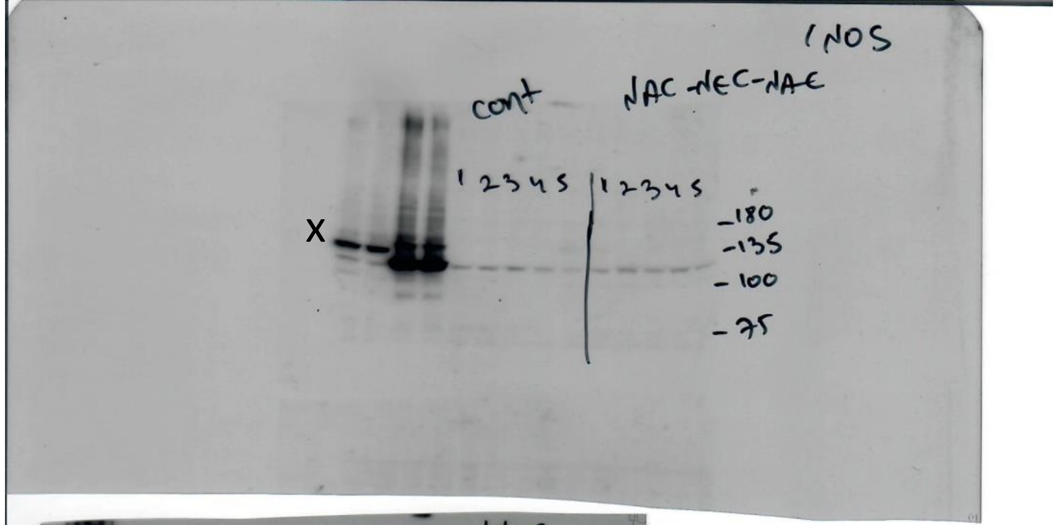

INOS
cont
NAC-NEC-NAC
1 2 3 4 5
1 2 3 4 5
x
180
135
100
75

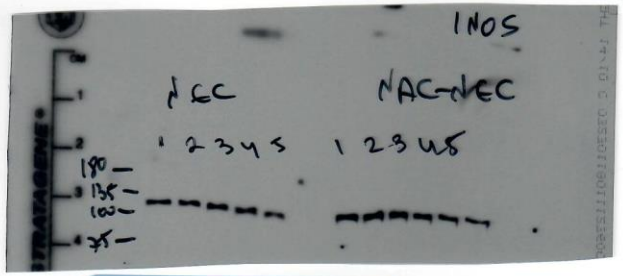

INOS
NEC
NAC-NEC
1 2 3 4 5
1 2 3 4 5
180
135
100
75

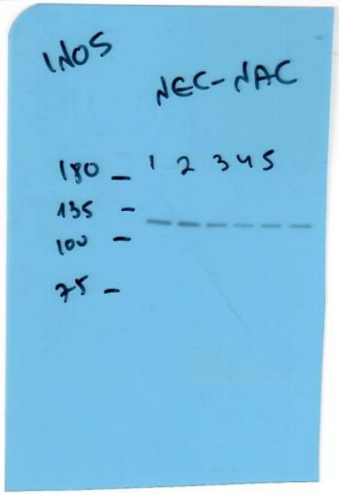

INOS
NEC-NAC
1 2 3 4 5
180
135
100
75

NFkb
FIG 4B

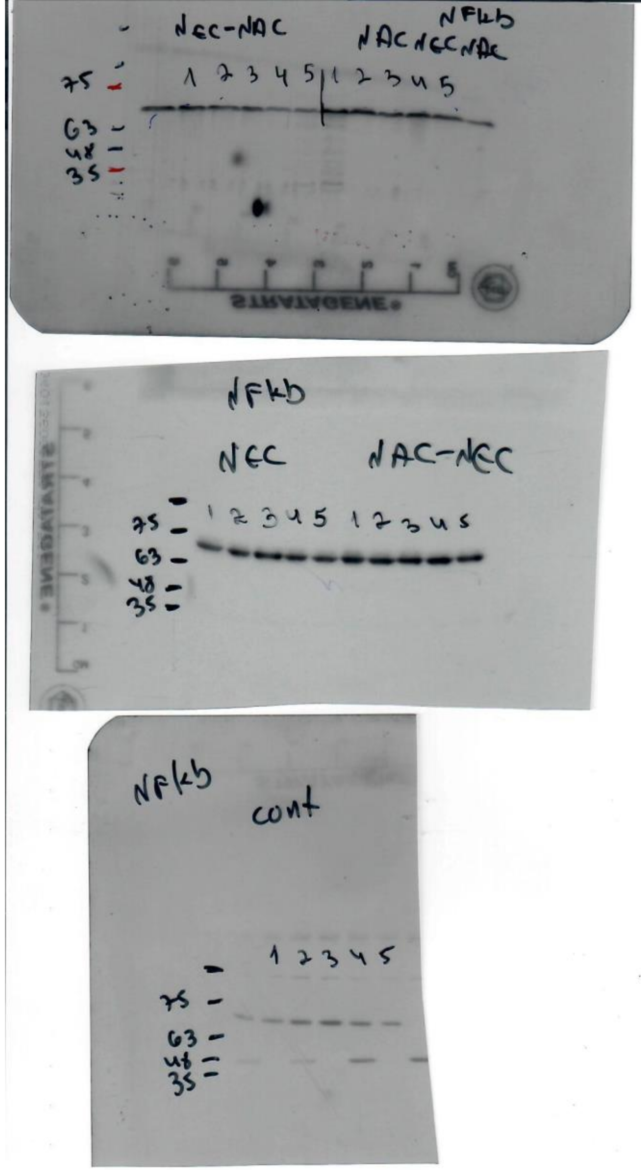
NEC-NAC
NFkb
NAC NEC NAC
1 2 3 4 5 1 2 3 4 5
75
63
48
35
STRATAGENE
NFkb
NEC
NAC-NEC
1 2 3 4 5 1 2 3 4 5
75
63
48
35
NFkb
cont
1 2 3 4 5
75
63
48
35

Crude data

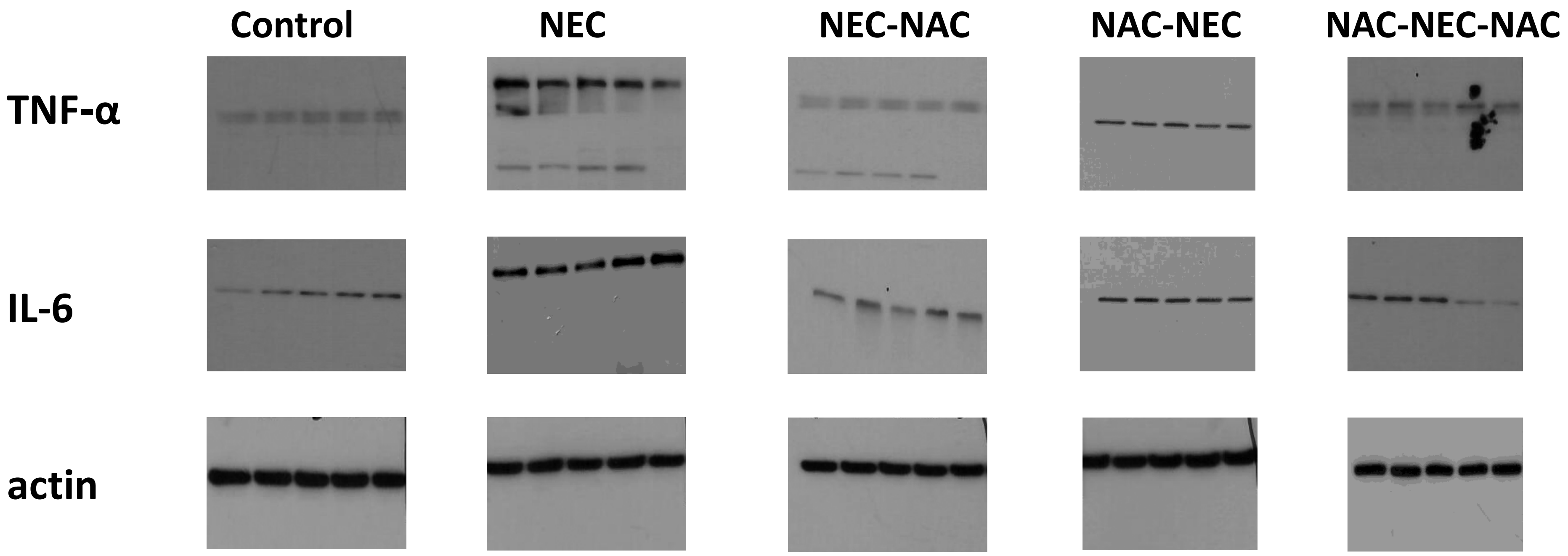

Crude data

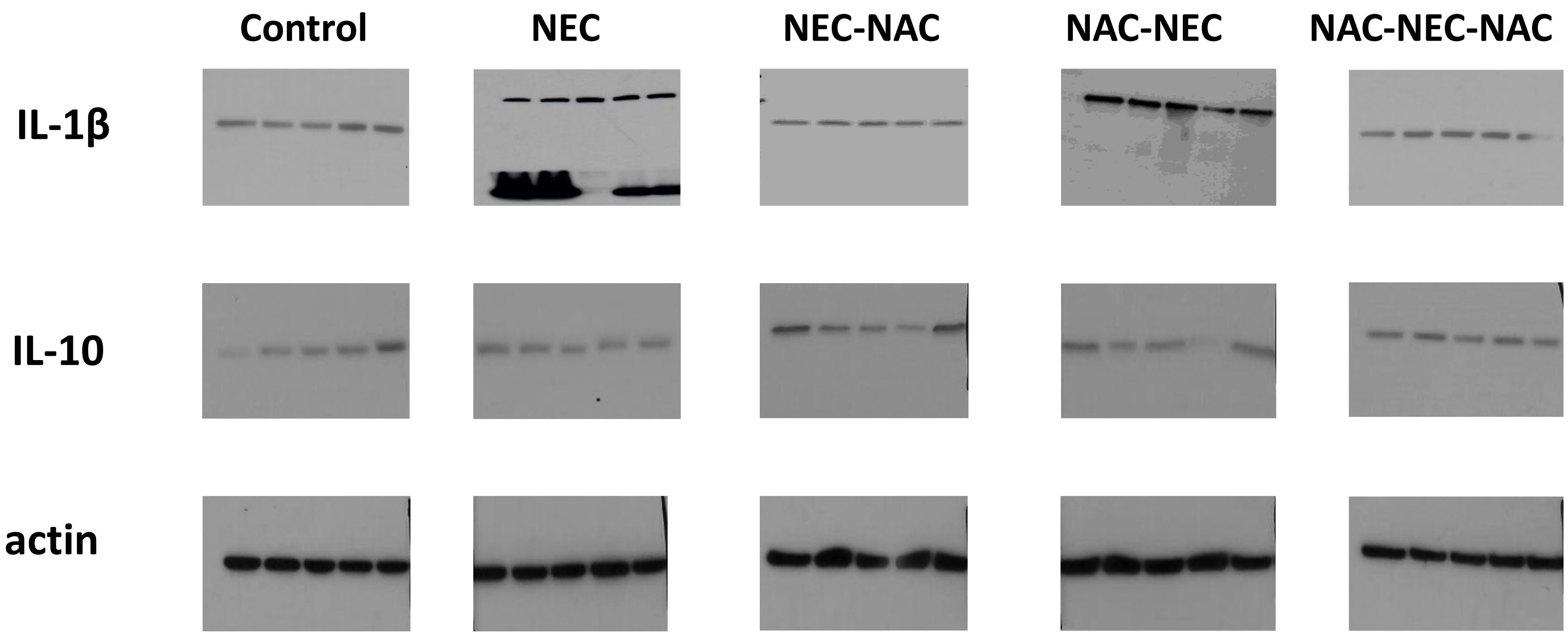

Crude data

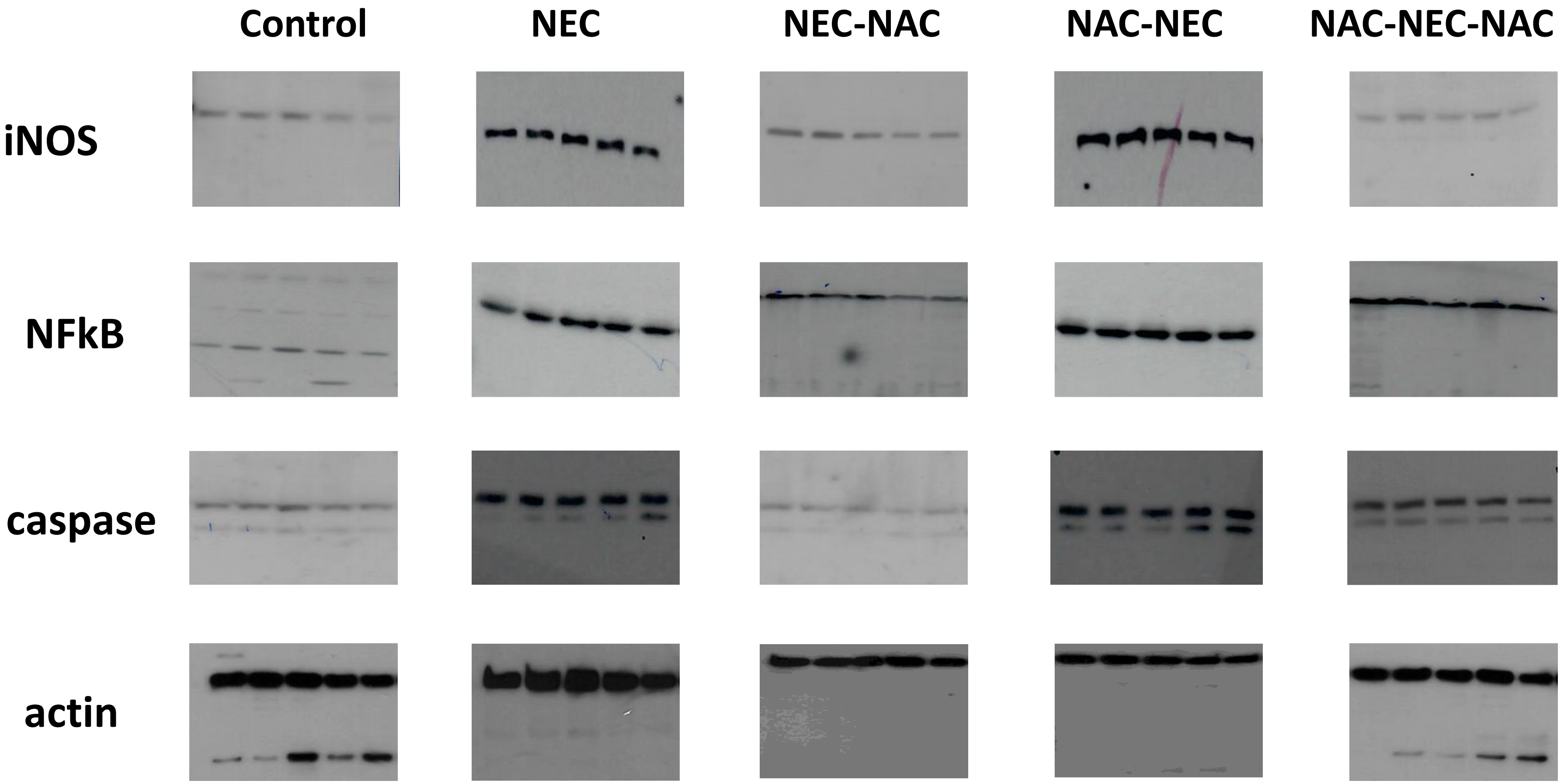
Control
NEC
NEC-NAC
NAC-NEC
NAC-NEC-NAC
iNOS
NFkB
caspase
actin
