# Supplementary material for: Prophylactic antenatal N-Acetyl Cysteine administration combined with postnatal administration can decrease mortality and injury markers associated with necrotizing enterocolitis in a rat model
Source: PLoS One. 2020 Jun 1;15(6):e0233612. doi: 10.1371/journal.pone.0233612 (PMC7263616; doi:10.1371/journal.pone.0233612)
Supplement: S1 Table — (DOCX) [file pone.0233612.s002.docx]

| Rat TNFα-F | AAAGCATGATCCGAGATGTG |
| --- | --- |
| Rat TNFα-R | AGCAGGAATGAGAAGAGGCT |
| Rat IL6-F | AAGAGACTTCCAGCCAGTTGCC |
| Rat IL6-R | ACTGGTCTGTTGTGGGTGGTATC |
| RAT iNOS-F | GACAACGTTCCTGTGGTCCT |
| RAT iNOS-R | TCCAGTGTGCTCTTCAGGTG |
| RAT NFKb-F | AACACTGCCGAGCTCAAGAT |
| RAT NFKb-R | ATCGGCTTGAGAAAAGGAG |
| RAT IL-10-F | GCAGGACTTTAAGGGTTACTTGG |
| RAT IL-10-R | GGGGAGAAATCGATGACAGC |
| RAT CASPASE 3-F | GTGGAACTGACGATGATATGGC |
| RAT CASPASE 3-R | CGCAAAGTGACTGGATGAACC |
| RAR Actin-F | GATATCGCTGCGCTCGTC |
| RAT Actin-R | TGGGGTACTTCAGGGTCAGG |
